# 2022 Outstanding Contributions to ISCB Award: Reinhard Schneider

**DOI:** 10.1093/bioinformatics/btac337

**Published:** 2022-06-27

**Authors:** Christina Fogg, Diane Kovats, Martin Vingron

**Affiliations:** Freelance Writer, Kensington, MD, USA; International Society for Computational Biology, Leesburg, VA, USA; Max Planck Institute for Molecular Genetics, Berlin, Germany

Each year, the Outstanding Contributions to ISCB Award recognizes an ISCB member for noteworthy service contributions toward the betterment of ISCB through exemplary leadership, education, and service. The 2022 Outstanding Contributions to ISCB Award recipient is Reinhard Schneider.


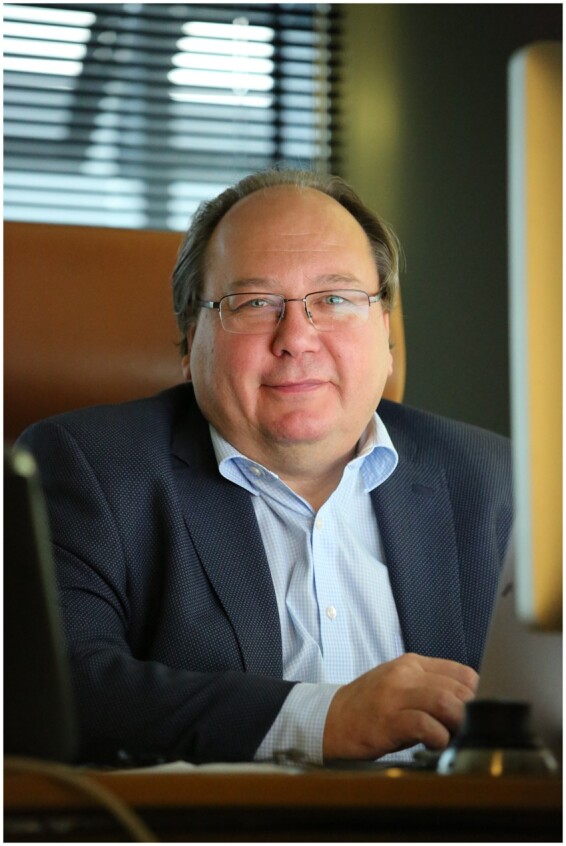


Reinhard Schneider is Full Professor in Bioinformatics, Head of Bioinformatics Core Facility, and Head of the ELIXIR Luxembourg Node at the University of Luxembourg. His research interests include developing and improving algorithms related to structure/function predictions of proteins. Schneider has devoted more than 20 years of service to ISCB in various capacities. He became an ISCB member in 1997 but became more involved as a co-organizer of ISMB with Thomas Lengauer in 1999. Schneider joined the ISCB Board of Directors in 2005 at a time when the Society was experiencing financial turmoil. His experiences working with startups helped him work with other board members to improve the management and financial stability of the organization. Schneider worked with Bettina Roth to redesign the ISCB web portal and improved features related to membership and registration, thus making the member portal user-friendly and reliable. Schneider introduced an option for members to purchase multi-year memberships, and he helped introduce other member benefits, and together these strategies boosted both membership and revenue.

Schneider served as ISCB Vice President from 2005 to 2009 and ISCB Treasurer from 2009 to 2016. His time as treasurer included developing an investment strategy for a portion of ISCB funds, which has given the Society greater financial stability. Beyond these roles, Schneider has served on various committees related to ISCB’s annual meetings, including ISMB/ECCB2007 (Vienna), ISMB2008 (Toronto), ISMB/ECCB2009 (Stockholm), ISMB2010 (Boston), ISMB/ECCB2011 (Vienna), ISMB2012 (Long Beach), ISMB/ECCB2013 (Berlin), ISMB2014 (Boston), and ISMB/ECCB2015 (Dublin). He has co-organized several international ISCB affiliated meetings in Africa, Asia, and Latin America, including ISCB Africa (2010: Bamako, Mali; 2011: Cape Town, South Africa) in cooperation with the African Society for Computational Biology and Bioinformatics (ASBCB), ISCB Latin America (2010: Montevideo, Uruguay; 2014: Belo Horizonte, Brazil), and most recently ISCB Asia (2011: Kuala Lumpur, Malaysia; 2012: Shen Zhen, China; 2013: Seoul, South Korea). His involvement with meeting organization includes helping launch and support the live coverage of ISMB via microblogging, making it one of the first life science conferences providing coverage in this manner. This initiative was started at ISMB2008 in Toronto and continues today.

Schneider is deeply gratified by his work in helping establish the ISCB Student Council (ISCBSC). He said, “It was great to see the enthusiasm of younger people getting involved in ISCB and to help them establish the ISCBSC with its own activities. I have invited very good students from developing nations to my lab through the ISCBSC internship initiative and hope these types of programs can help students launch their careers.”

Schneider is thankful for his diverse experiences with ISCB and encourages trainees to seek similar opportunities. He said, “It is important to learn skills that are outside of a typical graduate program, like management and organization skills, and how to work with others on different types of teams.” Schneider will be remembered as one of the ISCB’s leaders who helped financially secure the Society and broaden the ISCB membership. He will continue to support the ISCB community as a lifetime ISCB member.

